# One-Year Medication Treatment Patterns, Healthcare Resource Utilization, and Expenditures for Medicaid Patients with Schizophrenia Starting Oral Atypical Antipsychotic Medication

**DOI:** 10.1007/s10488-023-01327-1

**Published:** 2023-12-10

**Authors:** Kristin Richards, Michael Johnsrud, Christopher Zacker, Rahul Sasané

**Affiliations:** 1 TxCORE (Texas Center for Health Outcomes Research and Education), The University of Texas at Austin, 2409 University Avenue, Austin, TX 78712 USA; 2 Cerevel Therapeutics LLC, 222 Jacobs Street, Suite 200, Cambridge, MA 02141 USA

**Keywords:** Schizophrenia, Antipsychotic medication, Medicaid, Medication adherence

## Abstract

Oral atypical antipsychotic (OAAP) medications are the most commonly prescribed treatment for the management of schizophrenia symptoms. This retrospective study, using Medicaid claims data (2016–2020), followed patients for 12 months after initiating OAAP therapy. Study outcomes included OAAP adherence, switching, augmentation, healthcare resource utilization (HRU), and expenditures. All-cause and schizophrenia-related HRU and expenditures were compared between adherent and nonadherent cohorts. Among 13,007 included patients (39.1 ± 12.8 years of age, 57.0% male, 36.1% Black, 31.8% White, 9.7% Hispanic), 25.7% were adherent to OAAPs (proportion of days covered [PDC] ≥ 0.8). During the 1-year follow-up period, Black individuals were in possession of an OAAP for an average of 166 days compared to 198 and 202 days for White and Hispanic patients, respectively. Approximately 16% of patients switched OAAP medications and 3.2% augmented therapy with an OAAP added to their index medication. Nearly 40% of patients were hospitalized during follow-up and 68.4% had emergency department (ED) visits. A greater proportion of nonadherent patients had all-cause inpatient (41.7% vs. 34.1%, p < 0.001) and ED visits (71.7% vs. 58.8%, p < 0.001) compared to adherent patients. Annual total healthcare expenditures were $21,020 per patient; $3481 higher for adherent versus nonadherent patients. Inpatient expenditures comprised 44.6% and 30.6% of total expenditures for nonadherent and adherent patients, respectively. Hospitalized patients’ total expenditures were $23,261 higher compared to those without a hospitalization. Adherence to OAAP medication is suboptimal and associated with increased utilization of costly hospital and ED resources. Efforts to improve therapies and increase medication adherence could improve clinical and economic outcomes among individuals with schizophrenia.

## Introduction

Schizophrenia is a debilitating chronic condition affecting approximately 1% of the US population (Dixon et al., 2018). It is a complex and severe mental illness characterized by positive (e.g., delusions, hallucinations, thought disorders), negative (e.g., social withdrawal, lack of motivation, reduced ability to experience pleasure [anhedonia]), and cognitive symptoms (e.g., memory deficits, attention deficits) (Carbon & Correll, 2014; McCutcheon et al., 2020). Employment rates among people with schizophrenia are low (14–17%) (Rosenheck et al., 2006; Salkever et al., 2007) and many experience homelessness (~ 20%) (Folsom et al., 2005) and incarceration, with an estimated 2–6.5% prevalence of schizophrenia in correctional facilities (Pilon et al., 2020). Cloutier et al. (2016) estimated the US economic burden of schizophrenia in 2013 to be $155.7 billion, with direct healthcare costs of $37.7 billion (24.2% of total). Kadakia et al. (2022b) updated this estimate ($US2019) reporting an economic burden of $343.2 billion with direct healthcare costs of $62.3 billion (18.2% of total). Medication costs increased 19.2% over this 6-year period, while inpatient and outpatient medical costs increased by 80.9% and 58.8%, respectively.

The majority of non-institutionalized adults with schizophrenia in the US are insured through government healthcare programs, with 70% reporting Medicaid coverage based on 2014–2020 Medical Expenditure Panel Survey (MEPS) data (~ 35% with only Medicaid coverage and not dually eligible for Medicaid and Medicare) (Geissler et al., 2023). Only 4% reported no insurance and 43% and 19% reported Medicare and commercial insurance, respectively. Including patients eligible and not eligible for Medicare coverage, Pilon et al. (2021) estimated the prevalence of schizophrenia in Medicaid programs at 2.3–4.0%. They also reported a conservative estimate of $14,000 in higher annual healthcare costs for patients with schizophrenia compared to beneficiaries without the illness. Interest in treatment patterns, health outcomes, and costs in Medicaid patients with schizophrenia is high given that Medicaid programs provide healthcare coverage for a significant portion of this population.

Antipsychotic (AP) drugs are the first-line medication treatment for patients with schizophrenia to manage symptoms and reduce the risk of relapse. Medication nonadherence, however, is notably high in this population, with up to two-thirds of patients reported to be at least partially nonadherent to their AP medication (Kaplan et al., 2013). Long-acting injectable (LAI) APs, utilized by 13% of state Medicaid schizophrenia patients in 2018 (Patel et al., 2022), have demonstrated improved medication adherence (Greene et al., 2018; Kaplan et al., 2013). However, the mainstay of treatment for patients with schizophrenia is oral APs, specifically the newer generation (atypical) APs. Atypical APs are associated with less extrapyramidal symptoms (EPS) compared to first-generation (typical) AP medications (Divac et al., 2014). Nevertheless, adverse drug effects, including EPS, along with medication-related complications (e.g., weight gain, type 2 diabetes, sexual dysfunction, reduced bone mineral density) are frequent causes of atypical AP discontinuation (Fabrazzo et al., 2022). Approximately half of Medicaid patients diagnosed with schizophrenia (49%) do not take AP medication (Patel et al., 2022).

Given the debilitating and costly nature of schizophrenia and the prevalence of patients with schizophrenia in Medicaid programs, it is important to routinely assess current trends in treatment patterns, healthcare resource utilization (HRU), and costs associated with this severe mental illness. This study focuses on Medicaid patients diagnosed with schizophrenia who are newly prescribed oral atypical APs (OAAPs) and do not switch to LAI APs within the first year of therapy. The primary objective was to assess OAAP adherence and compare HRU and direct medical expenditures between adherent and nonadherent OAAP patients.

## Methods

### Study Design and Data Source

We used de-identified Medicaid administrative healthcare claims data representing a blinded multi-state sample of 344,751 patients with evidence of a schizophrenia diagnosis (International Classification of Disease, Tenth Revision (ICD-10): F20.xx) between January 1, 2016 and December 31, 2020. The data included claims from medical and pharmacy files and enrollment information was also utilized. Both fee-for-service and managed care delivery designs were represented in the data.

The index date was defined as the first prescription for an OAAP medication between July 1, 2016 and December 31, 2019 (the earliest and latest possible index dates, respectively). All patients meeting the inclusion criteria were followed for 12 months post-index. An incident user design was selected in order to establish a standardized observation period for all patients that begins at OAAP initiation. Baseline variables were derived from the 6-month pre-index period. This study was deemed exempt by The University of Texas at Austin Institutional Review Board.

### Inclusion Criteria

To be included in the study, the following criteria were required: (1) evidence of a paid OAAP medication claim between 7/1/16 and 12/31/19; (2) no evidence of typical or atypical AP medication utilization during the 6-month baseline period; (3) not dually eligible for Medicaid and Medicare pre- or post-index; (4) presence of a schizophrenia diagnosis associated with at least one inpatient or two outpatient claims during the pre- and/or post-index periods; (5) 18–63 years of age at index; (6) continuous Medicaid eligibility pre- and post-index; and (7) no evidence of LAI AP utilization.

### Study Variables

Baseline variables included patient age at index, sex, race/ethnicity and Quan-Charlson Comorbidity Index (CCI) score (Quan et al., 2005). Evidence of other severe mental illness was evaluated during the pre- and post-index periods. Outcome variables, based on the 12month observation period, consisted of total OAAP medication days, proportion of patients who added to or switched from the index OAAP, OAAP adherence, and all-cause and schizophrenia-related HRU and expenditures.

A measure of total medication days was calculated for each patient by summing the days of drug possession during the 1-year post-index period. This was done for both the index OAAP as well as for all OAAPs (treated as a class) dispensed during follow-up. In cases where augmented therapy (i.e., > 1 OAAP medication) was prescribed for a patient, each medication day still counted as a single day in the total medication days equation. Patients were identified as having switched from the index OAAP if they had a claim for another OAAP product and the index OAAP was not refilled. The new OAAP had to be dispensed within 30 days of the end of the index OAAP medication window. In cases were the index product was refilled and two OAAP drugs overlapped for at least 60 days, the patient was classified as having augmented OAAP therapy.

Adherence to OAAPs was calculated for the 1-year follow-up period using the proportion of days covered (PDC) formula: total medication days/(365 days - hospitalized days). Patients with a PDC of 0.8 or greater were classified as adherent. PDC was calculated for both the medication prescribed at index and for all OAAPs.

The numbers of all-cause and schizophrenia-related inpatient, ED, and outpatient visits/services were calculated to assess HRU outcomes. Hospital length of stay (LOS) was calculated using admission and discharge dates. Healthcare expenditures ($2020, USD) were analyzed for the following services: hospitalizations, ED visits, outpatient visits/services, and outpatient prescriptions. Schizophrenia-related HRU and expenditures required a diagnosis of schizophrenia to be associated with the claim and OAAPs comprised the group of schizophrenia-related outpatient prescriptions.

### Statistical Analyses

For univariate results, counts and percentages were reported for categorical variables, while means and standard deviations (SD) were reported for continuous variables. Baseline characteristics and OAAP treatment patterns were compared among index medication groups using chi-square and Kruskal–Wallis H tests. Chi-square and Mann Whitney Wilcoxon tests were used for baseline and outcome comparisons by adherence status (adherent = PDC ≥ 0.8; nonadherent = PDC < 0.8). The level of significance was set a priori at p < 0.05 and all analyses were performed using Stata Statistical Software, Release 17.

## Results

Out of a total of 344,751 Medicaid patients with at least one recorded schizophrenia diagnosis between 2016 and 2020, a sample of 16,346 patients was identified who met all but the final inclusion criterion (no evidence of LAI AP utilization) (Appendix 1). Of this group, 3339 (20.7%) were prescribed an LAI AP at some point during the follow-up period and, thus, were also excluded from the analysis. The final study sample consisted of 13,007 patients all newly started on a single OAAP product. The mean age was 39.1 years (sd = 12.8) at index, with 41.6% in the 18–34 age group. Males accounted for 57.0% of the sample and race/ethnicity characteristics were as follows: 36.1% Black, 31.8% White, 9.7% Hispanic, 1.8% Other, and 20.6% Unknown. Two-thirds of patients (66.6%) had evidence of: (1) bipolar disorder (39.2%), (2) major depressive disorder (26.5%), and/or (3) schizoaffective disorder (40.1%). Overall sample characteristics are detailed in Table 1 and are also presented by index medication.

**Table 1:** Sample characteristics overall and by index medication

	Overall N = 13,007	Quetiapine N = 3335 (25.6%)	Risperidone N = 3222 (24.8%)	Olanzapine N = 2557 (19.7%)	Aripiprazole N = 1779 (13.7%)
Males, n (%)	7411 (57.0)	1819 (54.5)	1895 (58.8)	1648 (64.5)	951 (53.5)
Mean age in years (SD) [median]	39.1 (12.8) [38]	41.1 (12.5) [41]	39.3 (13.1) [39]	38.3 (12.8) [37]	37.5 (13.2) [36]
Age group, n (%)					
18–34 years	5405 (41.6)	1167 (35.0)	1330 (41.3)	1156 (45.2)	834 (46.9)
35–44 years	2746 (21.1)	725 (21.7)	630 (19.6)	530 (20.7)	358 (20.1)
45–54 years	2768 (21.3)	809 (24.3)	709 (22.0)	495 (19.4)	313 (17.6)
55–63 years	2088 (16.1)	634 (19.0)	553 (17.2)	376 (14.7)	274 (15.4)
Race/ethnicity, n (%)					
Black	4697 (36.1)	1195 (35.8)	1311 (40.7)	890 (34.8)	625 (35.1)
White	4131 (31.8)	1077 (32.3)	880 (27.3)	799 (31.3)	628 (35.3)
Hispanic	1263 (9.7)	293 (8.8)	269 (8.4)	260 (10.2)	196 (11.0)
Other	239 (1.8)	46 (1.4)	56 (1.7)	51 (2.0)	40 (2.3)
Unknown	2677 (20.6)	724 (21.7)	706 (21.9)	557 (21.8)	290 (16.3)
Mean Quan-CCI score (SD) [median]	0.78 (1.58)	0.94 (1.71) [0]	0.78 (1.69) [0]	0.70 (1.55) [0]	0.74 (1.42) [0]
Quan-CCI score group, n (%)					
CCI = 0	8330 (64.0)	1935 (58.0)	2128 (66.1)	1768 (69.1)	1106 (62.2)
CCI = 1	2702 (20.8)	778 (23.3)	611 (19.0)	460 (18.0)	415 (23.3)
CCI = 2 +	1975 (15.2)	622 (18.7)	483 (15.0)	329 (12.9)	258 (14.5)
Other severe mental illness, n (%)					
Bipolar disorder	5099 (39.2)	1547 (46.4)	1027 (31.9)	973 (38.1)	696 (39.1)
Major depressive disorder	3445 (26.5)	1028 (30.8)	762 (23.7)	574 (22.5)	567 (31.9)
Schizoaffective disorder	4332 (40.1)	1118 (40.0)	1039 (39.1)	922 (43.2)	505 (34.6)

Other index OAAPs included asenapine, brexpiprazole, cariprazine, clozapine, iloperidone, lurasidone, paliperidone and ziprasidone

*CCI* Charlson comorbidity index

Chi-square and Kruskal–Wallis H tests showed statistically significant differences (p < 0.001) among index medication groups for all variables

### OAAP Treatment Patterns

Nearly 84% of patients were prescribed one of four OAAPs at index: quetiapine (25.6%), risperidone (24.8%), olanzapine (19.7%), and aripiprazole (13.7%) (Table 2). Other index OAAPs included lurasidone (5.1%), ziprasidone (4.3%), and paliperidone (3.0%), as well as asenapine, brexpiprazole, cariprazine, clozapine and iloperidone which were each prescribed to ≤ 1% of patients. Overall mean total medication days was 152.3 (sd = 114.8) for the index OAAP and 184.2 (sd = 114.1) for all OAAPs dispensed during follow-up. Patients starting on aripiprazole had slightly lower total medication days compared to the other index OAAP groups.

**Table 2:** Medication days, proportion who added/switched OAAPs and adherence by index medication

	Overall N = 13,007	Quetiapine N = 3335 (25.6%)	Risperidone N = 3222 (24.8%)	Olanzapine N = 2557 (19.7%)	Aripiprazole N = 1779 (13.7%)
Mean index OAAP days* (sd) [median]	152.3 (114.8) [120]	157.9 (113.4) [120]	151.5 (114.8) [120]	150.5 (116.2) [120]	144.1 (108.6) [112]
Mean all OAAP days (sd) [median]	184.2 (114.1) [153]	184.8 (113.5) [157]	182.2 (114.8) [150]	182.3 (114.6) [150]	177.0 (108.5) [150]
Added another OAAP*, n (%)	411 (3.2)	142 (4.3)	81 (2.5)	66 (2.6)	38 (2.1)
Switched to another OAAP, n (%)	2118 (16.3)	525 (15.7)	501 (15.6)	401 (15.7)	277 (15.6)
Mean PDC for index OAAP* (sd) [median]	0.42 (0.31) [0.33]	0.43 (0.31) [0.33]	0.42 (0.31) [0.33]	0.41 (0.32) [0.33]	0.40 (0.30) [0.31]
Adherent^a^ to index OAAP*, n (%)	2484 (19.1)	653 (19.6)	615 (19.1)	497 (19.4)	270 (15.2)
Mean PDC for all OAAPs* (sd) [median]	0.50 (0.31) [0.42]	0.52 (0.31) [0.43]	0.50 (0.31) [0.41]	0.50 (0.31) [0.41]	0.48 (0.30) [0.41]
Adherent^a^ to all OAAPs*, n (%)	3344 (25.7)	852 (25.6)	820 (25.5)	665 (26.0)	380 (21.4)

*OAAP* oral atypical antipsychotic, *PDC* proportion of days covered

Other index OAAPs included asenapine, brexpiprazole, cariprazine, clozapine, iloperidone, lurasidone, paliperidone, and ziprasidone

*Chi-square and Kruskal–Wallis H tests showed statistically significant differences (p < 0.05) among index medication groups

^a^Adherent: PDC ≥ 0.8

Approximately a third of patients (32.8%) had at least one claim for an OAAP other than their index OAAP at some point during the follow-up period. One in four (25.2%, n = 3279) had claims for a single additional OAAP product and 7.6% (n = 988) had claims for two or more additional OAAPs during the year following medication initiation. According to the switching and augmentation definitions previously mentioned, 16.3% switched from their index OAAP to another medication and 3.2% added another OAAP to their index medication. The proportion of patients switching OAAP therapy was similar among the top four index OAAPs (15.6–15.7%) and the proportion of those augmenting therapy ranged from 2.1 to 4.3%.

Mean PDC for the index OAAP was 42.3% (sd = 31.6%) and 19.1% of patients (n = 2484) were classified as adherent (PDC ≥ 0.8). Mean adherence increased to 50.4% (sd = 31.3%) when considering all OAAP medication claims for a patient during the follow-up period and 25.7% (n = 3344) were classified as adherent. Of the adherent patients, 48.7% (n = 1628) had access to OAAP medication every day of the year following OAAP initiation (PDC = 1), according to the claims data. Adherence results were consistent among the olanzapine, quetiapine, and risperidone index groups, and slightly lower for those starting on aripiprazole. The distribution of patients by PDC range was similar across index OAAP groups.

Adherent patients (PDC ≥ 0.8) were older than their nonadherent counterparts (40.0 vs. 38.8 years, p < 0.001) (Table 3). Almost 32% of patients 55 years of age and older at index were adherent compared to 24.5% of those less than 55 years. Patients’ reported racial/ethnic background was significantly different by adherence status (p < 0.001), though adherence was low overall. Approximately 30% of White and Hispanic patients were adherent to their OAAP medication regimen, while only 20% of Black patients were adherent. White and Hispanic patients were in possession of an OAAP medication for 198 (sd = 118) and 202 (sd = 115) days on average during the study period; a full month longer than Black patients (166 days; sd = 109).

**Table 3:** Sample characteristics by adherence status^a^

Sample characteristics	Overall N = 13,007	Adherent N = 3344; 25.7%	Nonadherent N = 9663; 74.3%	P value
Males, n (%)	7411 (57.0)	1894 (56.8)	5517 (57.1)	0.647
Mean age at index, yrs (SD) [median]	39.1 (12.8) [38]	40.0 (13.2) [40]	38.8 (12.7) [38]	** < 0.001**
Age group, n (col%) [row%]				** < 0.001**
18–34 years	5405 (41.6)	1325 (39.6) [24.5]	4080 (42.2) [75.5]	
35–44 years	2746 (21.1)	648 (19.4) [23.6]	2098 (21.7) [76.4]	
45–54 years	2768 (21.3)	705 (21.1) [25.5]	2063 (21.3) [74.5]	
55–63 years	2088 (16.1)	666 (20.0) [31.9]	1422 (14.7) [68.1]	
Race/Ethnicity, n (col%) [row%]				** < 0.001**
Black	4697 (36.1)	918 (27.5) [19.5]	3779 (39.1) [80.5]	
White	4131 (31.8)	1281 (38.3) [31.0]	2850 (29.5) [69.0]	
Hispanic	1263 (9.7)	385 (11.5) [30.5]	878 (9.1) [69.5]	
Other	239 (1.8)	73 (2.2) [30.5]	166 (1.7) [69.5]	
Unknown	2677 (20.6)	687 (20.5) [25.7]	1990 (20.6) [74.3]	
Mean Quan-CCI score (SD) [median]	0.78 (1.58) [0]	0.82 (1.62) [0]	0.77 (1.57) [0]	0.206
CCI = 0, n (%)	8330 (64.0)	2129 (63.7)	6201 (64.2)	0.599
CCI = 1, n (%)	2702 (20.8)	651 (19.5)	2051 (21.2)	**0.031**
CCI = 2 + , n (%)	1975 (15.2)	564 (16.9)	1411 (14.6)	**0.002**
Other severe mental illness, n (%)				
Bipolar disorder	5099 (39.2)	1188 (35.5)	3911 (40.5)	** < 0.001**
Major depressive disorder	3445 (26.5)	907 (27.1)	2538 (26.3)	0.332
Schizoaffective disorder	4332 (40.1)	1167 (34.9)	3165 (32.8)	** < 0.001**

*CCI* Charlson comorbidity index

Chi-square tests used for categorical variables; Mann Whitney Wilcoxon tests used for continuous variables

^a^Adherent: PDC ≥ 0.8 (based on post-index claims for all OAAPs)

### Healthcare Resource Utilization and Expenditures

Overall, 5175 patients (39.8%) were hospitalized during the 12-month observation period and 68.4% had ED visits (Table 4). A greater proportion of patients who were not adherent to their OAAP regimen had all-cause hospital (41.7% vs. 34.1%, p < 0.001) and ED visits (71.7% vs. 58.8%, p < 0.001) compared to adherent patients. Adherent patients, however, had significantly more all-cause outpatient medical visits/services compared to nonadherent patients (56.0 vs. 38.7, p < 0.001). These comparisons held for HRU associated with a schizophrenia diagnosis. Compared to adherent patients, a greater proportion of those who were nonadherent to OAAPs had schizophrenia-related inpatient (24.7% vs. 19.3%, p < 0.001) and ED visits (20.2% vs. 16.0%, p < 0.001), and nonadherent patients had fewer outpatient visits (8.1 vs. 14.2, p < 0.001).

**Table 4:** Healthcare resource use and expenditures by adherence status^a^

	Overall N = 13,007	Adherent N = 3344; 25.7%	Nonadherent N = 9663; 74.3%	P value
Healthcare resource utilization				
All-cause				
Patients with ≥ 1 visit, n (%)				
≥ 1 inpatient visit	5175 (39.8)	1141 (34.1)	4034 (41.7)	** < 0.001**
≥1 ED visit	8894 (68.4)	1965 (58.8)	6929 (71.7)	** < 0.001**
≥ 1 outpatient visit/service	12,902 (99.2)	3324 (99.4)	9578 (99.1)	0.117
Number of visits/days, mean (sd) [median]				
Inpatient visits	0.97 (2.04) [0]	0.86 (2.04) [0]	1.01 (2.04) [0]	** < 0.001**
Inpatient days ^b^	17.0 (22.3) [9]	18.9 (27.2) [10]	16.5 (20.7) [9]	0.350
ED visits	3.5 (6.4) [1]	2.8 (5.9) [1]	3.7 (6.6) [2]	** < 0.001**
Outpatient visits/services	43.2 (59.8) [23]	56.0 (68.5) [32]	38.7 (55.8) [20]	** < 0.001**
Schizophrenia-related^c^				
Patients with ≥ 1 visit, n (%)				
≥ 1 inpatient visit	3063 (23.3)	646 (19.3)	2390 (24.7)	** < 0.001**
≥ 1 ED visit	2487 (19.1)	536 (16.0)	1951 (20.2)	** < 0.001**
≥ 1 outpatient visit/service	8711 (67.0)	2442 (73.0)	6269 (64.9)	** < 0.001**
Number of visits/days, mean (sd) [median]				
Inpatient visits	0.54 (2.56) [0]	0.49 (3.67) [0]	0.55 (2.04) [0]	** < 0.001**
Inpatient days^b^	11.3 (14.3) [7]	12.2 (16.8) [8]	11.1 (13.6) [7]	**0.015**
ED visits	0.19 (0.39) [0]	0.16 (0.37) [0]	0.20 (0.40) [0]	** < 0.001**
Outpatient visits/services	9.6 (28.6) [2]	14.2 (35.9) [3]	8.1 (25.4) [1]	** < 0.001**
Expenditures ($2020, USD), mean (sd) [median]				
All-cause				
Inpatient expenditures	8518 (45,665) [0]	7222 (32,193) [0]	8967 (49,474) [0]	** < 0.001**
ED expenditures	1144 (2832) [257]	951 (2974) [127]	1210 (2778) [311]	** < 0.001**
Outpatient expenditures	6731 (10,802) [3398]	8476 (13,371) [4398]	6127 (9685) [3087]	** < 0.001**
Total medical expenditures	16,393 (48,758) [5833]	16,649 (36,989) [6535]	16,304 (52,218) [5592]	** < 0.001**
Prescription expenditures	4627 (15,637) [1150]	6957 (12,574) [2416]	3821 (16,408) [823]	** < 0.001**
Total expenditures	21,020 (51,828) [9397]	23,606 (39,775) [12,687]	20,125 (55,365) [8407]	** < 0.001**
Schizophrenia-related^c^				
Inpatient expenditures	2599 (14,959) [0]	2354 (14,177) [0]	2683 (15,220) [0]	** < 0.001**
ED expenditures	135 (594) [0]	106 (518) [0]	145 (618) [0]	** < 0.001**
Outpatient expenditures	1128 (3250) [160]	1553 (3972) [270]	982 (2946) [133]	** < 0.001**
Total medical expenditures	3862 (15,490) [361]	4013 (14,892) [456]	3810 (15,692) [348]	** < 0.001**
OAAP expenditures	1459 (3351) [163]	2580 (4779) [359]	1071 (2575) [111]	** < 0.001**
Total expenditures	5321 (15,757) [1235]	6593 (15,417) [1808]	4881 (15,850) [928]	** < 0.001**

*ED* emergency department, *PDC* proportion of days covered

X^2^ tests used for categorical variables; Mann Whitney Wilcoxon tests used for continuous variables

^a^Adherent: PDC ≥ 0.8 (based on post-index claims for all OAAPs)

^b^Calculated for patients with inpatient visits

^c^Schizophrenia diagnosis associated with medical claims

**Fig. 1:**
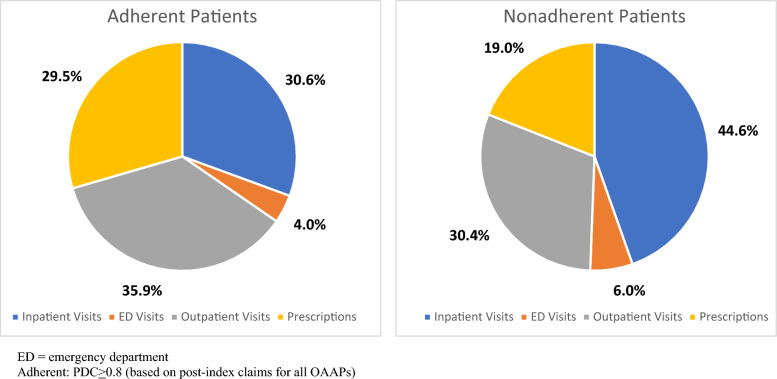
Expenditure proportions for patients adherent and nonadherent to oral atypical antipsychotic medications

Mean 12-month total healthcare expenditures per patient after OAAP initiation were $21,020 (sd = $51,828) overall; $3481 higher for adherent patients compared to their nonadherent counterparts (p < 0.001). Schizophrenia-related expenditures were $1712 higher for adherent patients (p < 0.001). Inpatient and ED expenditures accounted for half of total expenditures for nonadherent patients (44.6% and 6.0%, respectively) and a third of all expenditures for adherent patients (30.6% and 4.0%, respectively) (Fig. 1). However, outpatient visit and prescription expenditures accounted for a larger part of total expenditures for adherent patients (outpatient visits: 35.9% vs. 30.4%; prescriptions: 29.5% vs. 19.0%). As expected, expenditures for OAAP medications were higher (+ $1509) for adherent patients compared to those who were not adherent during the year following OAAP initiation. Regardless of adherence status, total expenditures were $23,261 higher on average for patients with at least one hospitalization compared to those who were not hospitalized ($33,464 vs. $10,203, respectively). Comparing patients with and without ED visits, total expenditures were higher for those with ED visits by an average of $11,789 ($23,186 vs. $11,397, respectively).

## Discussion

This analysis of OAAP treatment patterns serves as an updated examination of utilization and expenditure outcomes for Medicaid patients with schizophrenia who initiated oral AP treatment between 2016 and 2020. Although we focused on patients who did not switch to an LAI product during the first year of OAAP therapy, we found that 20.7% of otherwise eligible patients (meeting all but the final inclusion criterion) had at least one LAI AP claim later in the follow-up period—an increase from Marcus et al.’s and Patel et al.’s findings of 9% and 13%, respectively (derived from 2010 to 2013 and 2018 multi-state Medicaid claims data, respectively), evidencing this increasing trend (Marcus et al., 2015; Patel et al., 2022). The majority of those who currently take AP medications, however, use an oral dosage form.

As demonstrated in other AP adherence studies (Lieberman et al., 2005; Marcus et al., 2015; Shah et al., 2018), our sample of Medicaid patients with schizophrenia exhibited significantly suboptimal medication adherence. Overall, patients were in possession of their index OAAP for approximately 5 months out of the year, and this was only extended by a month when considering utilization of all OAAPs. The Clinical Antipsychotic Trials of Intervention Effectiveness (CATIE) randomized clinical trial showed discontinuation at 3.5 months, 4.6 months, 4.8 months, and 9.2 months for patients starting on ziprasidone, quetiapine, risperidone, and olanzapine, respectively (Lieberman et al., 2005). In the current study, approximately 20% of patients were adherent to their index medication (PDC ≥ 0.8) and 26% were adherent when considering all OAAP medications for which they had claims. Similarly, Manjelievskaia et al. and Pilon et al. found a 22.3% and 28.1% OAAP adherence rate, respectively, in their studies of Medicaid patients with schizophrenia (Manjelievskaia et al., 2018; Pilon et al., 2017b). These low proportions of adherent patients bolster concerns regarding schizophrenia patients’ ability to remain on therapy over time. We agree with Fabrazzo and colleagues on the “need for new treatments with improved tolerability and efficacy” (Fabrazzo et al., 2022). Unacceptably, the AP adherence disparity between Black and non-Black patients reported 20 years ago remains today (Herbeck et al., 2004; Mark et al., 2003). Effective medication adherence interventions are warranted, as well as programs that examine the broader non-medical drivers of health context contributing to medication nonadherence (Wilder et al., 2021).

Our findings indicate that 39.8% and 68.4% of patients had at least one all-cause inpatient or ED visit, respectively, with higher rates reported for nonadherent vs. adherent patients. Our results are similar to those of other studies that included OAAP cohorts of Medicaid patients with schizophrenia using claims data and a 12-month follow-up period (33.9–58.2% for inpatient visits; 56.5–65.8% for ED visits) (Manjelievskaia et al., 2018; Pilon et al., 2017a, 2017b). Our schizophrenia-related inpatient and ED rates (not reported in the above cited studies) were 23.3% and 19.1%, respectively. These may be conservative estimates as a schizophrenia diagnosis was required on the claim for the designation. Patients with schizophrenia often have multiple unique mental health diagnoses appearing in claims data, as we also observed in our study (Manjelievskaia et al., 2018).

Our results found the 12-month all-cause average direct medical spend after OAAP initiation to be $21,020 per patient, within the range of recent estimates of $16,000–$32,000 reported by others (Jiang & Ni, 2015; Kadakia et al., 2022a, 2022b; Pesa et al., 2017; Pilon et al., 2017b). Hospital and ED utilization and expenditures (all-cause and schizophrenia-related) were slightly higher for the nonadherent group—well-established associations with medication nonadherence in this population (Offord et al., 2013). Lack of medication adherence that leads to relapse and hospitalization puts additional strain on the nation’s healthcare system which currently has a shortage of psychiatric beds (American Psychiatric Association, 2022). Adherent patients’ hospital and ED expenditures, however, were not negligible and the reasons for these visits should be studied further. Adherent patients had higher utilization and expenditures for outpatient services and prescriptions, resulting in modestly higher total expenditures (+ $3500) compared to their nonadherent counterparts. Jiang and Ni (2015) found similar results when comparing adherent and nonadherent patients taking OAAPs (total costs were $4000 more for adherent patients). We hypothesize that those who consistently take their medications are more likely to be adherent with other aspects of their care, including seeing their providers and receiving outpatient services on a consistent basis. This is an area ripe for future study. Regardless of adherence status, patients with hospitalizations and/or ED visits had higher mean all-cause expenditures (by ~ $23,000 and ~ $12,000, respectively) than those without these visits, presumably indicating that schizophrenia relapse was avoided.

### Limitations

The primary strengths of this study include the recency of data, the large sample size, and the use of claims data from multiple US states. Drug exposures can be reliably identified using pharmacy claims (Lau et al., 1997). However, when using these data to assess medication adherence, the idea that patients begin taking their medication on the day of dispensing and follow the prescribing directions is an assumption. We have more certainty that a patient received a medication, but cannot verify consumption. Also, PDC is a proxy for adherence and the 80% adherence threshold, while often used in AP adherence research, has not been validated. We did not account for OAAP dose, which could impact results. We could not determine duration of illness as the date of first schizophrenia diagnosis was not available. We were unable to identify miscoded data fields and had no information on healthcare services outside of the Medicaid program. We recommend caution in the generalization of our results to the entire US Medicaid population as we were blinded to the number and identity of the states included in our dataset. There was no access to Medicaid program policies (e.g., prior authorization, formularies) which may impact outcomes.

It is worth noting that schizophrenia studies utilizing Medicaid claims data often encounter very large attrition rates as inclusion criteria are applied (Kadakia et al., 2022a; Manjelievskaia et al., 2018; Pilon et al., 2017a, 2017b). Our study was no different as our sample dropped 57.0% (N = 196,508) from a starting point of nearly 345,000 patients with a schizophrenia diagnosis between 2016 and 2020 to including only those with OAAP claims during the index identification period (N = 148,243) (Appendix 1). There were no prescription claim records in the data during the index identification period for 38% of these excluded patients, perhaps due to Medicaid-Medicare dual eligibility (Geissler et al., 2023). Some patients had evidence of typical AP (~ 4%) or atypical LAI AP (~ 11%) utilization only. The remaining 47% had prescription coverage, but claims for APs were not present in the data. Patient selection affects the generalizability of results and should be carefully considered.

## Conclusion

Following initiation of OAAPs among Medicaid patients with schizophrenia, 12-month medication adherence is suboptimal and contributes to increased inpatient and ED utilization and expenditures. Poor adherence to AP medication is prevalent in this group of patients and programs should encourage the identification of factors contributing to nonadherence among their members. Furthermore, differences that continue to exist between demographic factors, such as race/ethnicity, point towards potential areas of health inequity that must be addressed. Understanding these factors can be helpful in fostering policies based upon physician–patient shared decision-making by aligning treatment with patient needs and preferences. Finally, opportunity exists for the development of new and innovative therapies that represent improved treatment efficacy and limited side effect profiles that could positively impact adherence rates and resulting health outcomes.

## Data Availability

Not applicable.

## Appendix

See Table 5.

**Table 5:** Attrition

Inclusion criterion	Included patients (n)	Excluded patients (n)
Patients with schizophrenia diagnosis (ICD10: F20) Jan 2016–Dec 2020	344,751	
Patients with prescription claim records Jul 2016–Dec 2019	270,471	74,280
Patients with OAAP claims Jul 2016–Dec 2019	148,243	122,228
Patients with no pre-index AP claims	47,438	100,805
Patients not dually eligible for Medicaid and Medicare during 18 month study period	41,270	6168
Schizophrenia diagnosis during 18-month study period	29,969	11,301
18–63 years of age at index	26,764	3205
18 months of continuous Medicaid eligibility	16,346	10,418
Patients with no LAI AP claims	13,007	3339
Final sample	13,007	
